# Quantification of phosphoinositides reveals strong enrichment of PIP_2_ in HIV-1 compared to producer cell membranes

**DOI:** 10.1038/s41598-019-53939-z

**Published:** 2019-11-27

**Authors:** Frauke Mücksch, Mevlut Citir, Christian Lüchtenborg, Bärbel Glass, Alexis Traynor-Kaplan, Carsten Schultz, Britta Brügger, Hans-Georg Kräusslich

**Affiliations:** 1 Department of Infectious Diseases, Virology, University Hospital Heidelberg, Heidelberg, Germany; 2 Cell Biology & Biophysics Unit, European Molecular Biology Laboratory (EMBL), Heidelberg, Germany; 3 Heidelberg University Biochemistry Center (BZH), Heidelberg, Germany; 4 ATK Innovation, Analytics and Discovery, North Bend, WA 98045 USA; 5 Department of Medicine/Gastroenterology, University of Washington School of Medicine, Seattle, WA USA; 6 Department of Chemical Physiology and Biochemistry, Oregon Health and Science University (OHSU), Portland, OR USA; 7 German Center for Infectious Disease Research, partner site Heidelberg, Heidelberg, Germany

**Keywords:** Biochemistry, Microbiology

## Abstract

Human immunodeficiency virus type 1 (HIV-1) acquires its lipid envelope during budding from the plasma membrane of the host cell. Various studies indicated that HIV-1 membranes differ from producer cell plasma membranes, suggesting budding from specialized membrane microdomains. The phosphoinositide PI(4,5)P_2_ has been of particular interest since PI(4,5)P_2_ is needed to recruit the viral structural polyprotein Gag to the plasma membrane and thus facilitates viral morphogenesis. While there is evidence for an enrichment of PIP_2_ in HIV-1, fully quantitative analysis of all phosphoinositides remains technically challenging and therefore has not been reported, yet. Here, we present a comprehensive analysis of the lipid content of HIV-1 and of plasma membranes from infected and non-infected producer cells, resulting in a total of 478 quantified lipid compounds, including molecular species distribution of 25 different lipid classes. Quantitative analyses of phosphoinositides revealed strong enrichment of PIP_2_, but also of PIP_3_, in the viral compared to the producer cell plasma membrane. We calculated an average of ca. 8,000 PIP_2_ molecules per HIV-1 particle, three times more than Gag. We speculate that the high density of PIP_2_ at the HIV-1 assembly site is mediated by transient interactions with viral Gag polyproteins, facilitating PIP_2_ concentration in this microdomain. These results are consistent with our previous observation that PIP_2_ is not only required for recruiting, but also for stably maintaining Gag at the plasma membrane. We believe that this quantitative analysis of the molecular anatomy of the HIV-1 lipid envelope may serve as standard reference for future investigations.

## Introduction

Human immunodeficiency virus type 1 (HIV-1) assembles and buds at the host cell plasma membrane (PM) of infected cells. The main structural protein of HIV-1, Gag, mediates all of the essential events within this process, which is illustrated by the fact that Gag alone is sufficient to form virus-like particles (VLPs)^1^. Virus morphogenesis requires Gag trafficking to and association with the inner leaflet of the PM, where Gag assembles into a multimeric lattice comprising ca. 2,500 Gag molecules per viral particle^2^. Assembled particles are then released from the cell membrane by the ESCRT (endosomal sorting complexes required for transport) machinery, which is recruited in a Gag-dependent manner^2^. In order to form infectious virions, incorporation of additional viral components into nascent virions is required. They include the viral genome, replication enzymes and Env glycoproteins, which are all recruited to the assembly site by Gag^3^.

As HIV-1 buds from the PM of infected cells, the viral lipid envelope is derived from the cellular PM and it could therefore be assumed that it largely resembles the lipid composition of the latter. However, this is not the case. First reports stating differences between the lipid composition of HIV-1 and host cell PMs came from Aloia and colleagues. Using chromatography experiments, they showed that HIV-1 exhibits a significantly different phospholipid profile and fluidity than PMs of virus producing cells^4,5^. The notion of HIV-1 budding from specialized membrane domains received further support by the observation that Gag co-localized with *bona fide* marker proteins of nanodomains enriched in sphingolipids and cholesterol, and these proteins were also found in cell-free viral particles^6–9^.

Advances in lipid mass spectrometry (MS) allowed for more comprehensive and quantitative analysis of the HIV-1 lipidome. Using this approach, the lipidome of purified HIV-1 particles was compared to total membranes of producer cells^10^. Phosphatidylcholine (PC) and phosphatidylethanolamine (PE), the main phospholipids of mammalian membranes, were reduced in HIV-1 membranes. Conversely, sphingomyelin (SM), plasmalogen phosphatidylethanolamine (pl-PE) and phosphatidylserine (PS) were enriched in the viral membrane and an increase in saturated PC species was observed^10^. In agreement with the results obtained by chromatography experiments^5^, the cholesterol-to-phospholipid ratio in HIV-1 particles was increased about two-fold compared to producer cell membranes^10^. These observations were largely confirmed in subsequent lipid mass spectrometry analyses comparing HIV-1 and producer cell PM: SM, PS and saturated acyl chains were consistently increased in the viral membrane at the expense of PC and PE^11,12^. Cholesterol constitutes a major lipid in the plasma membrane, and was found to be further increased in some, but not all HIV-1 lipidome analyses^11,12^. Consistent with its lipid content, the HIV-1 membrane was found to exhibit a liquid-ordered (l_o_) state when probed with an environmentally sensitive dye^13^. Together, these findings led to the conclusion that HIV-1 buds from clustered nanodomains enriched in sphingolipids and cholesterol, which are either pre-existing and selectively targeted by Gag or are induced by Gag assembly. Viral membrane composition and fluidity appear to be functionally important since altering lipid content as well as applying membrane-active molecules affecting membrane fluidity were shown to interfere with HIV-1 infectivity^8,10,14–18^.

HIV-1 Gag membrane binding is mediated by its N-terminal MA (matrix) domain and depends on N-terminal myristoylation and a surface exposed patch of basic residues within MA, interacting with acidic phospholipids. This basic patch in MA is also required for specific Gag targeting to the PM and deletions or specific substitutions in MA led to Gag targeting to and particle assembly at intracellular membranes^19–24^. Specific Gag targeting to the PM further requires the PM-specific phosphoinositide phosphatidylinositol 4,5-bisphosphate (PI(4,5)P_2_): Depleting PI(4,5)P_2_ abolished HIV-1 Gag PM targeting and virus production, while increasing PI(4,5)P_2_ at intracellular membranes redirected Gag to those sites^25^. Consistently, several studies showed that the presence of PI(4,5)P_2_ enhanced binding of Gag-derived proteins to liposomes *in vitro*^26–30^. Recently, we have shown that PI(4,5)P_2_ is not only required for initial targeting of Gag to the PM, but is also needed to maintain the assembling Gag lattice at the PM^31^.

Despite their obvious importance for HIV-1 particle formation, we have only limited information about the phosphoinositide composition of HIV-1 in comparison with the host cell PM. There is evidence for an enrichment of PI(4,5)P_2_ in viral particles compared to the host cell PM^11^, but viral phosphoinositides have not been fully quantified and detailed information on the molecular species distribution of acyl chains in phosphoinositides is lacking. This is mainly due to significant technical challenges, which have largely prohibited quantitative and comprehensive mass spectrometry analysis of phosphoinositides in different membranes. First, phosphoinositides occur in low to very low abundance when compared to other lipids. PIP_3_ is one of the least abundant phosphoinositides and even all phosphorylated forms of PI together (PIP, PIP_2_ and PIP_3_) make up less than 1% of the cellular lipid cohort^32–35^. Second, other lipids interfere with the detection of phosphoinositides during analysis. Finally, phosphoinositides yield only low levels of detectable ions in the mass spectrometry process due to their high phosphorylation state. In addition to these technical challenges, phosphoinositides are highly dynamic lipids, being rapidly converted into one another by cellular kinases and phosphatases.

Here, we made use of an optimized mass spectrometry workflow which allowed detection of the phosphoinositides PIP, PIP_2_ and PIP_3_ from whole cells, isolated PMs and purified HIV-1. This was made possible by optimized lipid extraction protocols combined with permethylation^36^, permitting quantification of all major classes of phosphoinositides and analysis of their molecular species profiles. Together with quantification of all other major lipid classes and of viral Gag protein and RNA content, this report provides the currently most comprehensive picture of the HIV-1 lipidome and its relation to the producer cell plasma membrane.

## Results

### Plasma membrane isolation from HIV-1 infected and uninfected MT-4 cells

**Figure 1:**
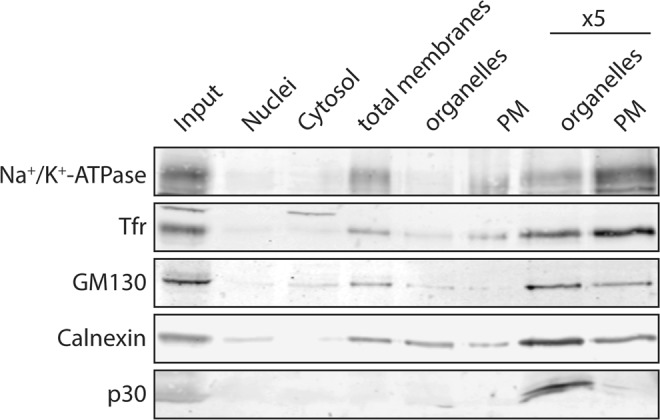
PM isolation by differential centrifugation and density centrifugation. Western blot analysis of MT-4 membrane fractions. Uninfected MT-4 cells were harvested, washed with PBS and subjected to PM isolation. 3% of each fraction (or 15% in lanes marked with “x5”) were loaded. The membrane was probed with antibodies against marker proteins for PM (Na^+^/K^+^-ATPase and transferrin receptor (Tfr)), Golgi apparatus (GM130), ER (Calnexin) and mitochondria (p30) in three consecutive rounds (up to two antibodies, from different species, per round). For improved clarity and conciseness, cropped areas of the blot are shown. All cropped regions originate from the same blot.

For comprehensive lipidome analysis, PMs were isolated from uninfected and infected MT-4 cells. Infected MT-4 cultures were >75% infected, as assessed by HIV-1 CA (capsid protein) expression (Supplementary Fig. S1A). Since we were specifically interested in phosphoinositides, which are highly dynamic and sensitive to physical membrane alterations^37^, we selected a rapid method for PM isolation that does not actively alter the cell surface rather than applying the previously used blebbing or silica bead-based PM isolation methods^11,12^. PMs were separated from other cellular constituents by mechanical disruption of cells followed by a combination of differential and density centrifugations. Equal amounts of the different fractions obtained relative to input material were subjected to Western Blot analysis and the loss of organelle marker proteins within the different fractions was evaluated (Fig. 1). To assess the purity of the PM fraction, we determined enrichment of PM and removal of non-PM membranes by probing input and PM fractions with antibodies against marker proteins indicative of the presence of PM (sodium potassium ATPase (Na + /K + ATPase), TfR), endoplasmic reticulum (ER; calnexin), Golgi (GM130) and mitochondria (p30). Na^+^/K^+^ ATPase and Tfr were clearly enriched in the PM fraction when compared to organelle membrane fractions, while the abundance of marker proteins for Golgi, ER and mitochondria was decreased in the PM fraction. To quantify these differences, we measured band intensities of the Western Blot shown in Fig. 1 and calculated the ratio between one of the PM markers (Na^+^/K^+^ ATPase or Tfr) and each individual organelle marker (ER/Golgi/mitochondria) in the input and PM fractions. The two different PM markers were enriched 1.8-fold (+/− 0.02) compared to an ER marker in the PM fraction, while Golgi markers were decreased fourfold (+/− 0.03) and mitochondria markers were decreased 14-fold (+/− 0.12) relative to PM markers in PM isolations compared to input. This analysis indicated strong PM enrichment, while residual ER- and a lower amount of Golgi membranes appear to be retained in the isolated PM fraction. ER contamination of PM isolations may cause some underestimation of PM-specific lipids in the PM, including e.g. phosphoinositides. Importantly, this method allowed isolation of PM with sufficient purity in a comparatively short period of time (the complete procedure takes less than 2 hours) at 4 °C.

### Virus preparation and lipid extraction

HIV-1 particles were purified from the medium of infected MT-4 cells by velocity gradient centrifugation on Optiprep gradients. This protocol yielded largely vesicle-free virus preparations^38^. Silver-stained gels of purified particles produced from MT-4 cells showed the Gag-derived CA and MA proteins as major HIV-1 particle constituents and confirmed the purity of the preparations and complete maturation (Supplementary Fig. S1B). Virus stocks were further quantitatively analyzed for HIV-1 CA and genomic RNA content (Supplementary Fig. S1C), and infectivity was titrated on susceptible target cells. These results allowed calculating the viral particle concentration in the respective samples and provided the basis for the subsequent estimation of viral lipid species on a per particle basis.

Standard lipid extraction according to Bligh and Dyer was applied as described^39^ to recover all lipid species (except phosphoinositides) directly from whole cells, isolated PMs and virus preparations.

For phosphoinositide analyses, samples were TCA precipitated and then sequentially subjected to single-phase neutral and acidic extractions as shown in the schematic in Supplementary Fig. S2A. Internal standards were spiked into both extracts. Subsequently, both extracts were processed by two-phase acidic extraction (Supplementary Fig. S2A). Lipids from neutral and acidic extracts were separately collected, derivatized, measured, and normalized based on the internal standards (standard curves for phosphoinositide quantitation are shown in Supplementary Fig. S2B–D). Finally, the normalized values from both extracts were summed up to yield the total level of the respective lipid in the original sample (Supplementary Fig. S2E). This multistep procedure was necessary for quantitative recovery of the different phosphoinositides: while 70% of PI was observed in the neutral extract, PIPs were mainly present in the acidic extract and PIP_3_ was exclusively recovered from the acidic extract (Supplementary Fig. S2E), as had been observed for radioactively labeled phosphoinositides^40^.

Purified PM and virus samples contain little biological material compared to whole cell samples, which decreases the precipitation efficiency by TCA, and also the reproducibility of the two-step lipid extraction procedure due to lack of a visible pellet prior to neutral extraction. Therefore, we tested bovine serum albumin (BSA) and poly-D-lysine (PL) for their suitability to be used as a carrier during TCA precipitation of the purified PM and virus samples. For this purpose, we analyzed the recovery of PIP and PIP_2_ in neutral and acidic extracts in the presence of BSA or PL by thin layer chromatography (TLC). PIP or PIP_2_ standards were transferred into test tubes and dried, PL or BSA was added and the samples were recovered by TCA precipitation. Pellets were subjected to neutral and acidic extraction and extracts were run on TLC and stained with iodine vapors. In the presence of BSA, PIP and PIP_2_ were lost from acidic extracts, since BSA remained soluble in the single-phase neutral extraction and precipitated at the interphase during two-phase extraction, leading to retention of PIP and PIP_2_ in the neutral extract that contains the bulk of membrane lipids (Supplementary Fig. S2F, BSA-samples). However, PL did not dissolve in the neutral extraction solvent and therefore turned out to be a suitable carrier with which we observed good recovery of PIP and PIP_2_ in acidic and neutral extracts (Supplementary Fig. S2F, PL-samples and S2G).

Phosphoinositides purified by lipid extraction without further modification have low ionization efficiency due to their highly acidic nature, and thus yield large amounts of ambiguous ions during electrospray ionization. To improve their ionization efficiency, we employed a derivatization technique using TMS-diazomethane to methylate phosphate groups of phosphoinositides as previously described^36,41^. This neutralizes the phosphoinositides and therefore improves their ionization. The derivatized phosphoinositides are also more stable and volatile than their free acid forms^36^. This procedure enabled us to resolve the neutralized phosphoinositides based on their fatty acid chain composition during liquid chromatography.

Accordingly, isolated PMs and virus preparations were TCA-precipitated in the presence of PL for phosphoinositide analysis and subsequently subjected to two-step neutral and acidic lipid extraction, followed by permethylation. Lipid extracts from purified HIV-1, from isolated PM fractions from uninfected or HIV-1 infected MT-4 cells or from complete membrane fractions of uninfected or HIV-1 infected MT-4 cells were subjected to nano-electrospray ionization or liquid chromatography/electrospray ionization tandem mass spectrometry. Samples were analyzed for the different cellular lipid classes and lipid species. This led to quantitative determination of a total of 478 individual lipids, for the first time including the molecular species distributions of all three phosphoinositide classes.

### Lipid composition of purified virus and producer cell membranes

Comparison of the lipid composition and species distribution between whole cell extracts from uninfected and infected MT-4 cells revealed no major differences (see Supplementary Table 1 and Supplementary Fig. S3), consistent with previous reports suggesting that HIV-1 infection does not alter the overall lipid profile of target cells.

**Figure 2:**
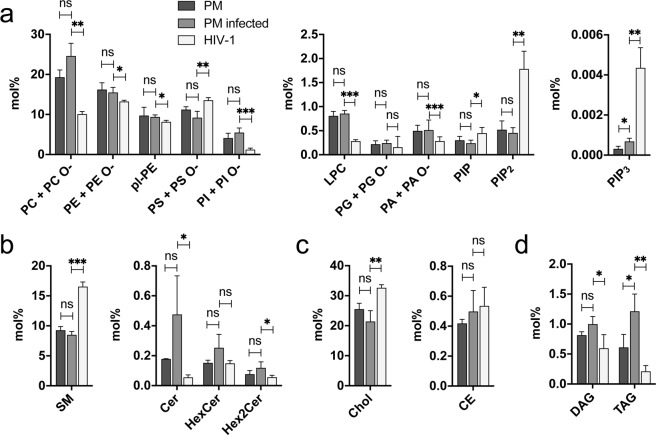
Lipid composition of PMs from uninfected or HIV-1 infected MT-4 cells and of the HIV-1 membrane. Lipid composition (in mol% of total lipids) of PMs isolated from uninfected (dark grey) or HIV-1 infected (medium grey) MT-4 cells and of HIV-1 particles produced in MT-4 cells (light grey). Shown are phospholipids (**a**) with an expanded scale for minor lipid species in middle and right panels, sphingolipids (**b**) and sterols (**c**) with an expanded scale for minor lipids in right panels, and glycerolipids (**d**). Data represent mean values and standard deviation of n = 4 independent virus productions and n = 3 independent PM purifications. Statistical significance was assessed with the two-tailed unpaired Student’s t-test; ***p≤0.001, **p≤0.01, *p≤0.1. PE O-, PS O-, PI O-, PG O-, and PA O- species contain either an ether linked alcohol or an odd-chain fatty acyl moiety.

In general, we also did not observe significant differences when comparing the lipid composition of PMs from uninfected and HIV-1 infected MT-4 cells. The only exceptions were PIP_3_ and triacylglycerol (TAG) levels, which were increased about 2-fold in the PMs of infected cells (Fig. 2A, right panel and 2D). Since TAG is not a membrane lipid it can be assumed that TAG amounts were caused by contamination with non-bilayer lipids.

Figure 2A shows that the phospholipid composition of HIV-1 membranes differs significantly from the host cell PM, as reported in previous studies^5,12^. PC, PE and PI levels were decreased in viral particles compared to host cell PM; the most abundant membrane phospholipid PC was reduced by half from ca. 20 mol% to ca. 10 mol% and PI levels were reduced even further from 5.2 mol% in the PM to 1.0 mol% in the viral membrane. In addition, the minor phospholipids lyso-PC (LPC) and phosphatidic acid (PA) were also reduced in the viral compared to the host cell PM; these two lipids had not been quantified in previous studies of HIV-1 membranes. PS was significantly enriched in HIV-1 when compared to the host cell PM as previously reported^5,12^. All phosphoinositides were detected in significantly higher amounts in viral particles when compared to the host cell PM (Fig. 2A, middle and right panel, further discussed below). Also, sphingolipids differed between viral particles and PM isolations (Fig. 2B). While SM was highly enriched in HIV-1 particles, as reported^5,10–12^, ceramide (Cer) was strongly decreased. Dihexosylceramide (Hex2Cer) showed a slight decrease in HIV-1 particles. Consistent with HIV-1 budding from nanodomains enriched in sphingolipids and cholesterol, we found cholesterol to be clearly enriched in HIV-1 membranes when compared to PMs of infected and non-infected cells (Fig. 2C), as reported before^5,11,12^.

**Figure 3:**
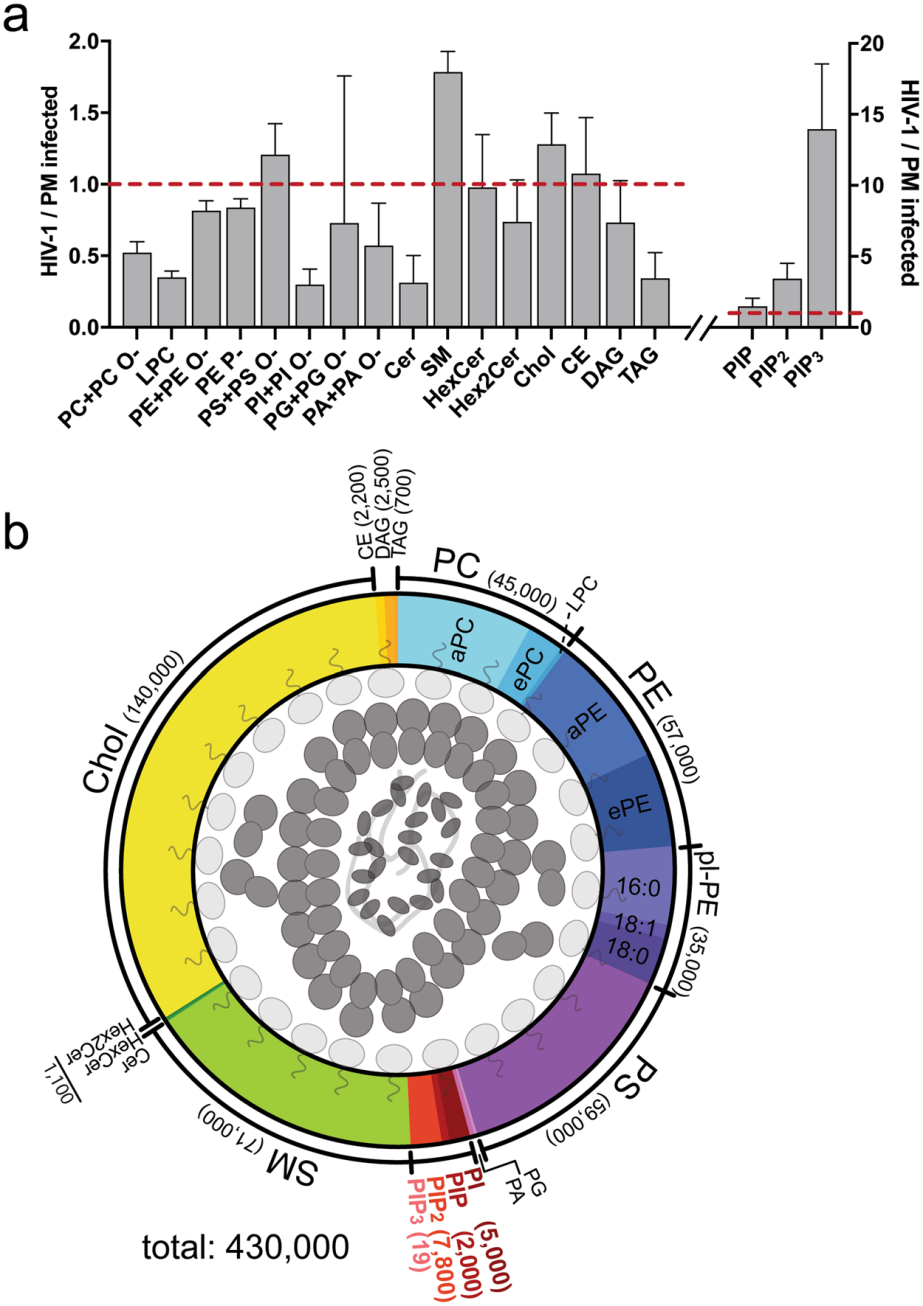
Differential enrichment of lipids in HIV-1 membranes. (**a**) Factors of enrichment of individual lipid classes in HIV-1 over PMs from infected MT-4 cells. Equal amounts (enrichment factor 1) are indicated by the broken red lines. Phosphoinositides are shown on a different scale. SM: sum of SM and dihydroSM species. Error bars represent standard deviation calculated from standard deviations of n = 3 independent PM isolations and n = 4 independent virus isolations. (**b**) Illustration of the lipid composition of a single average HIV-1 particle. We calculated a total number of 430,000 lipid molecules per average HIV-1 particle. Colored areas represent the relative contribution of different lipid classes (as indicated) to the overall HIV-1 lipidome. Molecule numbers for individual lipid classes including standard deviation from n = 4 independent virus preparations are given in Supplementary Table 2.

The lipids with the greatest enrichment in viral particles when compared to host cell PMs were PS, SM, cholesterol and the phosphoinositides (Fig. 3A). PS was increased 1.2-fold, SM 1.8-fold, cholesterol 1.3-fold and PIP 1.5-fold. By far, the most pronounced enrichment detected was for PIP_2_ (3.4-fold) and PIP_3_ (14-fold). PC (0.5-fold), LPC (0.3-fold), PI (0.3-fold), PA (0.6-fold), Cer (0.3-fold) and TAG (0.3-fold) were the lipids that showed the strongest decrease when comparing HIV-1 with PM from infected cells.

Within the phosphoinositol-containing lipids, the relative contributions changed from 83% PI, 6% PIP, 11% PIP_2_ and 0.01% PIP_3_ in the PM to 36% PI, 13% PIP, 51% PIP_2_ and 0.13% PIP_3_ in HIV-1 membranes, illustrating the dramatic alterations within this group of lipids (Fig. 3A).

To determine the molecular composition of the HIV lipid envelope, we normalized the lipid molecule numbers to the number of viral particles present in the extract, which was calculated from the copy number of genomic viral RNA (Supplementary Fig. S1C). This calculation yielded estimates for the total lipid content and for the number of molecules of different lipid classes in the membrane of an average HIV-1 particle. We calculated 430,000 lipid molecules per average virion based on four independent virus preparations. A graphical representation of the lipid composition of HIV-1 particles and numbers of respective lipid molecules per average HIV-1 particle for the various lipid classes are shown in Fig. 3B and Supplementary Table 2. We estimated approx. 2,000 PIP molecules, 7,800 PIP_2_ molecules and 20 PIP_3_ molecules in the lipid envelope per average HIV-1 particle. All three phosphoinositide classes were enriched when compared to the PM of HIV-1 infected MT-4 cells. Even though the absolute number of PIP_3_ molecules per virion is low, PIP_3_ is highly enriched in the HIV-1 lipid envelope as compared to the donor PM (Fig. 2A).

**Figure 4:**
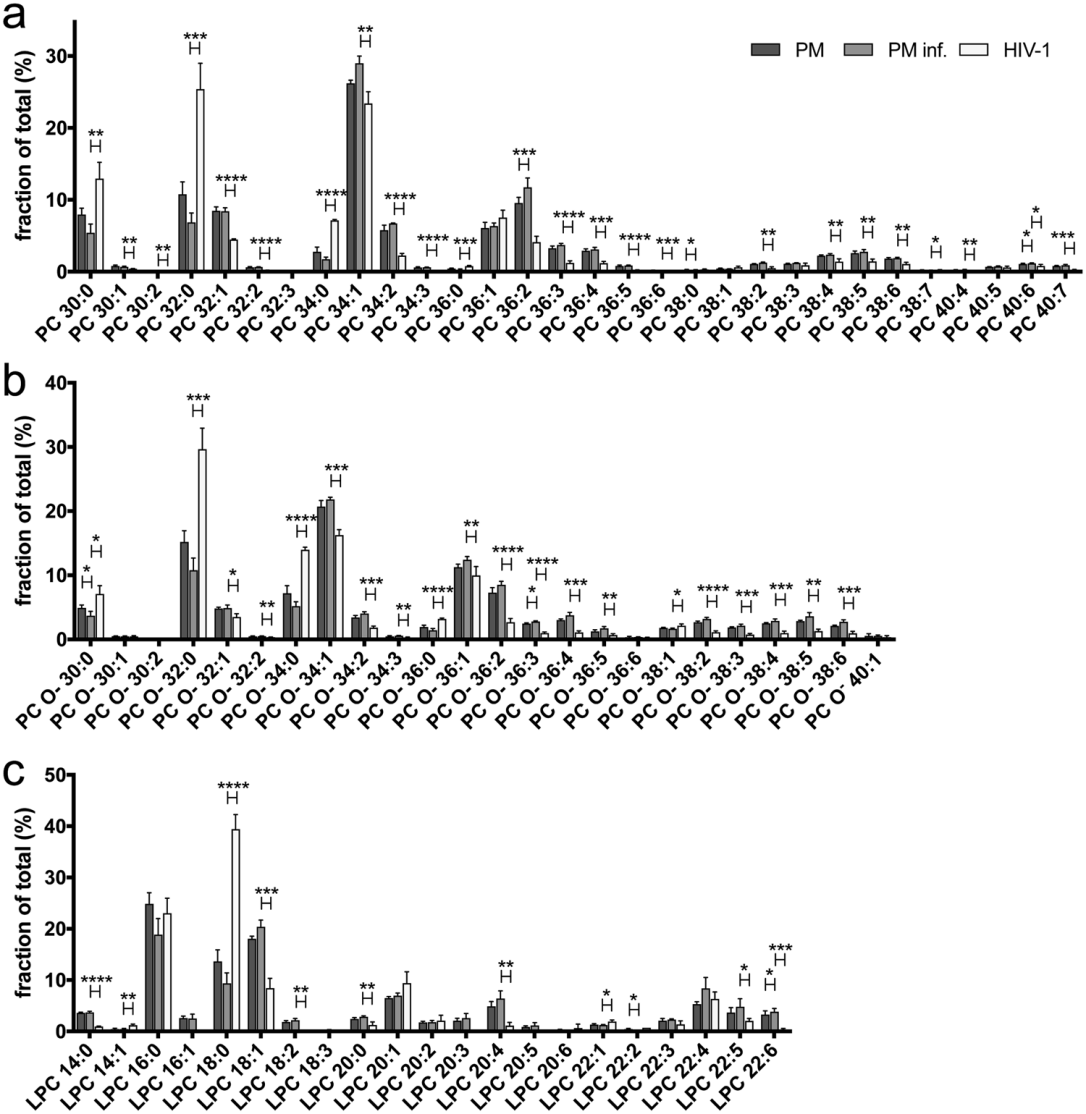
Molecular species distribution of phosphatidylcholine (PC). Quantitative lipid analysis of PM isolations from uninfected (PM) and HIV-1 infected (PM inf.) MT-4 cells and of viral membranes (HIV-1) was performed as described in Materials and Methods. Molecular species distribution of PC **(a)**, ether- or odd chain fatty acyl containing PC (PC O^−^) (**b**) and lyso-PC (LPC) (**c**) is given as fraction of total. Data represent mean values and standard deviation of n = 3 (PM isolations) or n = 4 (virus purifications) independent experiments. Statistical significance was assessed with the two-tailed paired (PM vs. PM inf.) or unpaired (PM inf. vs. HIV-1) Student’s t-test; ****p≤0.0001, ***p≤0.001, **p≤0.01, *p≤0.1 (only statistically significant differences are indicated).

We then determined the molecular species distribution of all phospholipids analyzed. Generally, acyl chain distributions were very similar in lipids from the viral membrane and from the producer cell PM (Supplementary Figs. S4–9). An exception were the molecular species of PC, lysoPC (LPC) and PI, as described before^12^. PC, LPC and PI were among the most strongly reduced lipids in the viral membrane (Fig. 3A). Compared with MT-4-derived PMs, we additionally observed a clear shift towards fully saturated acyl and ether PC species at the expense of mono- und poly-unsaturated PC species (Fig. 4A,B).

This shift was most pronounced for the shorter 30:0, 32:0 and 34:0 PC species. LPC also showed a strong enrichment of fully saturated species with short acyl chains at the expense of mono- and polyunsaturated LPC species (Fig. 4C).

For PI, we observed a clear shift towards short-chain PI species at the expense of longer ones and a reduction of polyunsaturated PI species with an increase of saturated and mono-unsaturated PI species (Supplementary Fig. S4A). In addition to what was observed before, we detected a shift towards shorter and less unsaturated species of acyl and ether/odd-chain fatty acyl-containing PG species in viral particles when compared to PMs of uninfected or infected cells (Supplementary Fig. S4C,D). PA, while showing a largely similar species distribution in HIV-1 membranes and PM from infected cells, showed some differences in PMs of non-infected cells, as those were enriched in 32:0, 32:1 and 34:1 PA at the expense of 36:1 PA when compared to HIV-1 particles and their producer cell PMs (Supplementary Fig. S5A). Except for a slight increase in PE 34:1, PE 36:1 and PS 36:1 species in HIV-1 membranes, the species distribution of PE, pl-PE, PS, SM, HexCer, Hex2Cer, Cer, and CE resembled that of PMs from infected or uninfected MT4 cells (see Supplementary Figs. S6–S9).

**Figure 5:**
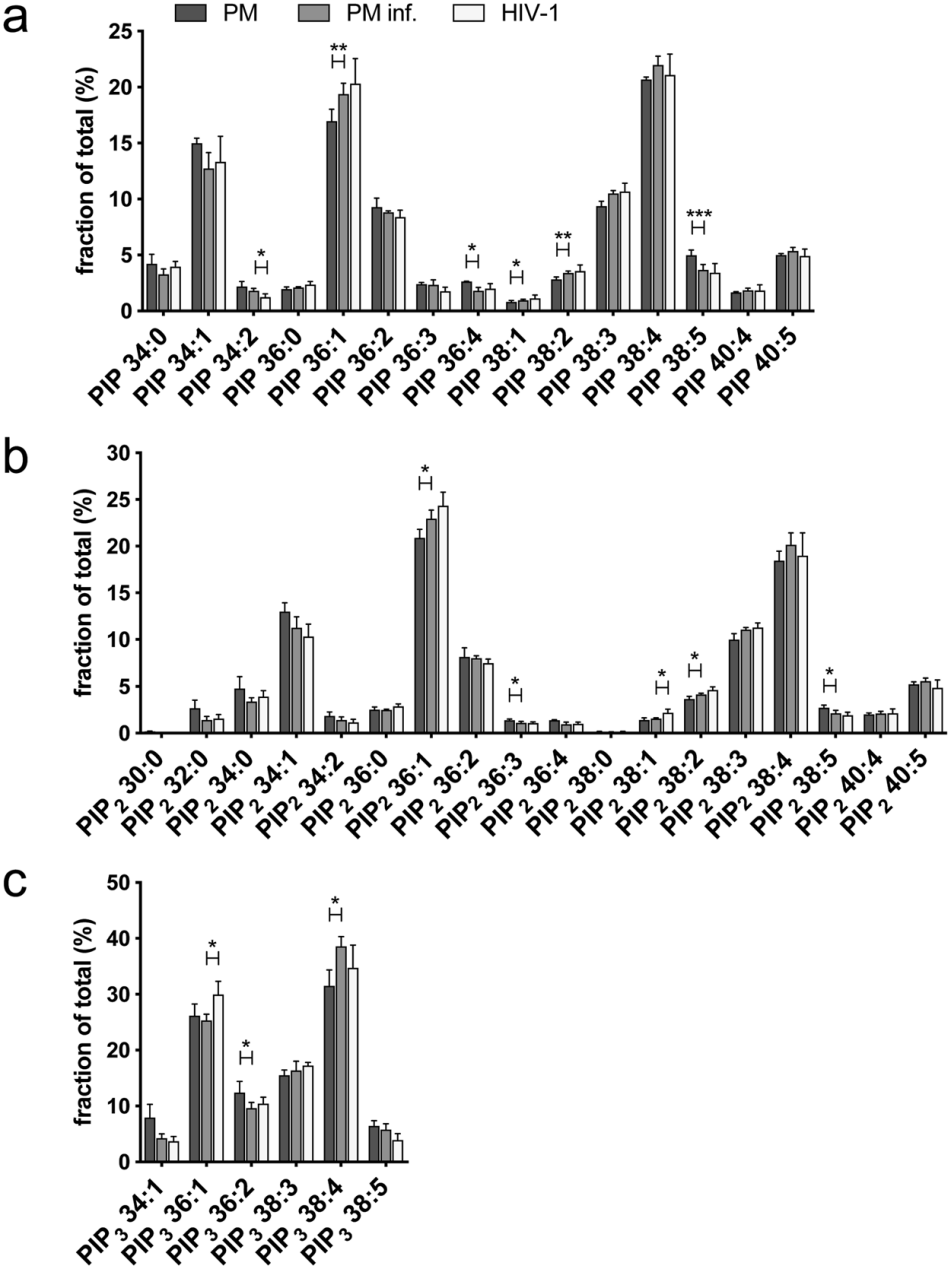
Molecular species distribution of phosphoinositides. Molecular species distribution of PIP (**a**), PIP_2_ (**b**) and PIP_3_ (**c**) extracted from uninfected (PM) and infected (PM inf.) MT-4 cells and from viral membranes (HIV-1) is given as fraction of total. Data represent mean values and standard deviation of n = 3 (PM isolations) or n = 4 (virus purifications) independent experiments. Statistical significance was assessed with the two-tailed paired (PM vs. PM inf.) or unpaired (PM inf. vs. HIV-1) Student’s t-test; ****p≤0.0001, ***p≤0.001, **p≤0.01, *p≤0.1 (only statistically significant differences are indicated).

The molecular species distribution of phosphoinositides was also largely similar in HIV-1 membranes and PMs of producer cells (Fig. 5) and when comparing uninfected and infected MT-4 cells (Supplementary Table 1). The most abundant molecular species of PIP, PIP_2_ and PIP_3_ were the polyunsaturated 38:4 species and the monounsaturated 36:1 species (Fig. 5), showing that the metabolism of phosphoinositides is highly interconnected. Longer acyl chain species such as 36:1 were enriched in PIP_2_ and PIP_3_ species in the HIV-1 membrane compared to PM at the expense of shorter acyl chain species. Taking into account the elevation of PIP_2_ levels in released virions, the viral membrane contains 4.2 times more PIP_2_ 36:1 than PM from infected cells (0.44 mol% vs. 0.1 mol%).

## Discussion

Here, we present a comprehensive, quantitative lipidome analysis of HIV-1 in comparison with isolated host cell PMs from HIV-1 infected and uninfected cells. We detected and quantified 25 different lipid classes, covering the vast majority of the membrane lipid spectrum, including in total 478 molecular lipid species. Thus, this work significantly expands the coverage of lipid species described previously by us and others^5,10–12^ and provides a quantitative analysis of all phosphoinositides, including PIP_3_, a minor phosphoinositide, which was not quantified in HIV-1 or PMs before. Based on this analysis, we present approximate numbers for lipid classes including phosphoinositides per average HIV-1 particle.

Our comprehensive analysis of the phosphoinositide content of plasma membranes isolated from HIV-1 infected cells and of HIV-1 particles derived thereof revealed a strong enrichment of PIP_2_ and PIP_3_ in viral membranes. The enrichment of PIP_2_ is in line with previously published data^11^ and the specific role of this lipid during HIV-1 assembly. As PI(4,5)P_2_ is the most abundant phosphoinositide in mammalian cells and comprises >99% of the PIP_2_ pool in mammalian plasma membranes^37,42^, PI(4,5)P_2_ is most likely the dominating isomer enriched in the viral particle as well. PI(4,5)P_2_ has been shown to be essential for PM targeting of HIV-1 Gag and thus for virus assembly and release^24,26,31^. Replenishing PI(4,5)P_2_ in PI(4,5)P_2_ depleted PM led to rapid induction of Gag assembly at the PM and production of infectious virus^31^. Taken together with structural analyses revealing a PI(4,5)P_2_ binding site in the MA domain of HIV-1 Gag, one might therefore assume roughly stoichiometric amounts of Gag polyproteins and PI(4,5)P_2_ molecules in HIV-1 particles. This is clearly not the case, however. Using quantitative lipid mass spectrometry combined with estimation of HIV-1 particle number in analytes, we determined the number of PIP_2_ molecules per average HIV-1 particle to be 7,834 ±2885. This translates into a more than threefold molar excess of PI(4,5)P_2_ over the ca. 2,500 Gag molecules per average HIV-1 particle. As the immature Gag layer does not completely cover the inner surface of the viral membrane^43^, Gag recruitment of PI(4,5)P_2_ may be even stronger. Alternatively, Gag-free zones could also accommodate PI(4,5)P_2_ clusters, but this could not explain the strong molar excess of PI(4,5)P_2_ over Gag in the viral membrane.

A strong enrichment of PIP_2_ in viral membranes is in line with our previous observation that PI(4,5)P_2_ is needed to maintain the nascent Gag lattice at the PM during later stages of assembly, since partially assembled Gag structures were lost from the PM upon PI(4,5)P_2_ depletion^31^. This result suggested a dynamic equilibrium between membrane-inserted and MA-buried myristate moieties with concomitant transient interactions of Gag with PI(4,5)P_2_ molecules at the assembly site since stable insertion of a large number of Gag-linked myristates into the membrane bilayer would be expected to retain the assembling Gag lattice at the membrane. Accordingly, several recent reports indicated that multiple surfaces on MA interact transiently and dynamically with one or more PI(4,5)P_2_ head groups^30,44^. Retaining PI(4,5)P_2_ in the viral assembly domain by these recurrent, transient Gag interactions would explain the observed high PI(4,5)P_2_ concentration in the virion. Given that PI(4,5)P_2_ is by far the most prominent phosphoinositide in the viral membrane, there appears to be no further metabolic conversion once PI(4,5)P_2_ is confined in the budding virion.

HIV-1 membranes were enriched in cholesterol, PS and SM at the expense of PC, PE and PI. These results are in good agreement with previously published HIV-1 lipidomics data^10–12^. Contrary to our previous studies^10,12^, pl-PE was not enriched in viral particles, and the observed slight decrease in pl-PE (0.9-fold when compared to PM from infected cells) was similar to the report by Chan and colleagues^11^. These authors also reported an increase in cholesterol in the HIV-1 membrane in comparison to PM from infected cells^11^, which was confirmed here. The observed minor differences between the different lipidomics studies may have been caused by differences in cell types and membrane isolation methods. While Lorizate *et al*. and Chan *et al*.^11,12^ used cationic colloidal silica beads to extract PMs, we used density gradient centrifugation. This method should not interfere with host cell metabolism, as we do not apply any chemical stimulation or alteration of the cell surface.

All studies of HIV-1 lipidomes reported a major increase of the outer leaflet lipid SM in the viral membrane^10–12^. In the current study, SM was enriched almost twofold in viral particles compared to PM from virus-producing cells. Since SM is restricted to the outer leaflet and Gag is a peripheral membrane-binding protein associating with the inner PM leaflet, Gag cannot directly recruit outer leaflet lipids into the HIV-1 assembly site. Obvious candidates for mediating Gag-dependent recruitment of SM in the outer leaflet would be inner leaflet lipids directly interacting with Gag or recruited by Gag into the assembly domain. Trans-bilayer coupling of inner and outer leaflet lipids has been reported to mediate actin dependent clustering of outer leaflet lipids and was shown to depend on acyl chain length of the respective lipids^45^. In this case, trans-bilayer coupling depended on PS 18:1/18:0 (i.e. 36:1), while molecular species of PS with shorter acyl chains could not mediate this effect^45^. Obvious candidates for inner leaflet lipids to couple outer leaflet lipids with HIV-1 Gag assembly would be PI(4,5)P_2_ and PS, both of which were found to be enriched in viral membranes and to be recruited by Gag. HIV-1 particles showed fourfold enrichment in PIP_2_ 36:1 (0.44 mol% in particles versus 0.10% in infected PMs). In addition, a slight but significant 1.3-fold increase of PS 36:1 was observed in virions when compared to PMs of infected cells. These results are consistent with the reported involvement of PS 36:1 in coupling outer leaflet lipid clustering to cytoplasmic signaling^46^, and would suggest a similar mechanism of trans-bilayer coupling for Gag-induced PS 36:1 and possibly PIP_2_ 36:1 clustering. Even though we detected a 16-fold increase of PIP_3_ 36:1 in virions compared to producer cell PM, the levels of 36:1 PIP_3_ in HIV-1 particles (0.0013 mol%) remain very low and a significant contribution to trans-bilayer coupling therefore seems less likely. Conceivably, the Gag-linked myristate group could also contribute to trans-bilayer coupling as has been observed for dimyristoylphosphatidylcholine in membrane bilayers^47^. However, in the case of HIV-1 Gag the relatively short myristate acyl chain (C14) is stably anchored on the Gag protein and is thus unlikely to reach sufficiently deep into the bilayer.

In summary, our results are consistent with HIV-1 budding from sphingolipid and cholesterol enriched nanodomains. The observed strong enrichment of PIP_2_ in the viral membrane supports our previously reported hypothesis of transient Gag interactions with PM PI(4,5)P_2_, and concomitant flipping of myristate between the inner leaflet of the PM and a lipid-binding pocket in the MA domain of Gag^31^. PIP_2_ and possibly also PS recruitment may then mediate accumulation of outer leaflet SM, thus rigidifying the viral membrane. Budding of HIV-1 from domains rich in sphingolipids and cholesterol might facilitate the fission step that releases viral particles from the plasma membrane by a mechanism that involves a tension gradient between more ordered domains in the viral bud and less ordered domains in the neck area^48^. This model would suggest Gag to be the master-regulator of the assembly membrane, directly or indirectly recruiting specific lipid species. While the alternative model of Gag targeting to pre-existing lipid microdomains of the observed composition and arranged by a different membrane regulator appears less likely, it is not excluded by the current results. Advances in multi-color super-resolution microscopy may in the future allow directly visualizing lipids and proteins at HIV-1 assembly sites, thus shedding light on this important issue.

## Materials and Methods

### Reagents

All chemicals and reagents were purchased from commercial sources unless otherwise noted.

Plasmid pNL4-3 was described previously^49^.

### Cell culture and virus purification

MT-4 cells^50^ (CVCL_2632; received from V. Bosch (DKFZ Heidelberg) in 1990 and passaged in the laboratory since) were cultured at 37 °C and 5% CO_2_ in Roswell Park Memorial Institute Medium 1640 (RPMI; Invitrogen). Medium was supplemented with 10% fetal calf serum (FCS; Biochrom), 100 U/ml penicillin and 100 µg/ml streptomycin and 5 mM HEPES.

For virus production, MT-4 cells^50^ were infected with HIV-1 strain NL4–3^49^, and the virus was harvested from co-cultures of infected and uninfected cells before cytopathic effects were observed as described in^38^. HIV-1 purification was performed as described^38^. Briefly, particles were concentrated from cleared media by centrifugation through a cushion of 20% (w/v) sucrose in PBS. Concentrated HIV-1 was further purified by velocity gradient centrifugation on an Optiprep gradient (Axis-Shield, Oslo, Norway). The visible virus band was collected and pelleted yielding an 1,800-fold concentration compared with the initial volume. Purity was assessed by separation of particles by SDS-PAGE (12.5% acrylamide) and subsequent silver-staining. Virus production was quantitated by an in-house enzyme-linked immunosorbent assay (ELISA) detecting the HIV-1 CA protein p24^51^. Virus titers were determined by evaluation of RNA copy number with PCR, as follows.

### Analysis of HIV-1 RNA copy number

Number of viruses in concentrated virus stocks was estimated by analysis of RNA copy number. For this, virus stocks were diluted 1:10 with PBS and lysed with 2x lysis buffer (50 mM KCl, 100 mM Tris-HCl, 40% Glycerol, 0.25% Triton X-100; pH 7.4). Subsequently, lysed samples were stepwise diluted further with PBS to a final dilution of 1:10,000,000–1:40,000,000. 600 µl of the dilutions were subjected to quantitative PCR using the Abbott RealTime HIV-1 assay REF 6L^18^ (Abbott Molecular Inc., Des Plaines, IL, USA) and the assay was performed according to the manufacturer’s instructions. The Abbott RealTime HIV-1 assay is able to quantitate HIV-1 over the range of 40 to 10,000,000 copies/ml and diluted samples measured here were in the range of approximately 60,000 to 200,000 copies/ml.

### Plasma membrane isolation and whole cell harvesting

PMs of uninfected and infected MT-4 cells were isolated using the Minute Plasma Membrane Protein Isolation Kit (Invent Biotechnologies, Blymouth, MA, USA) according to the Manufacturer’s instructions. For this, cells were harvested at time points when minimum 75% of cells were p24 positive, as assessed by flow cytometry. 4*10^7^ uninfected and infected MT-4 cells each were harvested per experiment and PM isolation was performed on four columns per sample (1*10^7^ cells/column).

Half of the resulting material was further used for general lipidomics and the other half for phosphoinositide lipidomic analysis. For this, samples were resuspended in PBS (general lipidomics) or water (phosphoinositide lipidomics). Samples used for phosphoinositide analysis were gently mixed with delipidated poly-D-lysine at a final concentration of 1 mg/ml. Afterwards, TCA was added to a final concentration of 10% (w/v), samples were vortexed for 30 s and incubated on ice for 15 min. Finally, samples were centrifuged at 20.000 g for 3 min at 4 °C, supernatant was discarded, and samples were frozen at − 80 °C

Whole cells were harvested at the same time, washed with PBS and directly frozen at – 80 °C as cell pellets (general lipidomics) or TCA precipitated (phosphoinositide lipidomics) with 10% ice-cold TCA in deionized water (as above but in the absence of poly-D-lysine), pelleted and frozen at – 80 °C.

### Flow cytometry

To analyze the amount of productively infected MT-4 cells, cells were washed and fixed in 4% PFA for 90 min, washed twice with PBS, and spun down. Pellets were incubated with HIV Gag p24 flow cytometry antibody KC57-FITC (PN 6604665, Beckman Coulter, Fullerton, CA, USA) at 1:50 dilution in PBS supplemented with 0.1% Triton X-100 and 0.1 mg/ml BSA for 30 min at RT. Subsequently, cells were washed twice, and analyzed using an FACS Verse flow cytometer (BD Biosciences, Franklin Lakes, NJ, USA). Unstained cells were used as a control for gating. Results were analyzed with the software FlowJo v10 (FlowJo, LLC, Ashland, OR, USA).

### SDS-PAGE and western blot

Samples for SDS-PAGE were lysed and boiled (95 °C, 10 min) in Lämmli buffer. Lysates were separated by SDS polyacrylamide gel electrophoresis (PAGE) and proteins were transferred to a methanol-activated Immobilon-FL polyvinylidene fluoride (PVDF) membrane (Merck Millipore, Burlington, MA, USA).

Sodium potassium ATPase (Na + /K + -ATPase), transferrin receptor (Tfr), GM130, Calnexin and p30 (mitochondria marker) were detected by probing the membrane with polyclonal anti-Na + /K + -ATPase clone EP1845Y (RRID:AB_1310695; Abcam, Cambridge, UK), monoclonal anti-Tfr clone H68.4 (RRID:AB_2533030; Thermo Scientific, Waltham, MA, USA), monoclonal anti-GM130 clone 35/GM130 (RRID:AB_398142; BD Biosciences, Franklin Lakes, NJ, USA), polyclonal anti-Calnexin (RRID:AB_11178981; Enzo Life Sciences GmbH, Lörrach, Germany) and polyclonal anti-p30 (kindly donated by W. Just, BZH, Heidelberg) antibodies, respectively, followed by secondary antibodies coupled to IRDye 800 or IRDye 680 (RRIDs:AB_621847 and AB_621845; LI-COR Biosciences, Lincoln, NE, USA). Fluorescent signals were detected using a LI-COR Odyssey CLx scanning system. To quantify band intensities, blots were analyzed using the Odyssey Image Studio v5.2 software (LI-COR Biosciences, Lincoln, NE, USA).

### Sample preparation prior to lipid extraction

All generated samples were split for general lipidomic and phosphoinositide measurements.

For general lipidomic analyses, cell pellets, PM isolations in PBS and purified HIV-1 in PBS were added to an excess of MeOH and subsequently subjected to lipid extraction.

For phosphoinositide lipidomic analyses, purified HIV-1 in PBS was mixed with 1 mg/ml delipidated poly-D-lysine and subjected to ice-cold TCA (10% (w/v) final concentration). TCA-treated cell pellets, PM isolations and HIV-1 particles were washed 2 x with 5% TCA + 10 mM EDTA. Subsequently, all samples were subjected to lipid extraction.

### Lipid extraction and analysis of general lipids

Cells, PM and viral particles were subjected to lipid extractions using an acidic Bligh & Dyer, except from plasmalogens, which were extracted under neutral conditions^52^. Lipid standards were added prior to extractions, using a master mix containing phosphatidylcholine (13:0/13:0, 14:0/14:0, 20:0/20:0; 21:0/21:0; Avanti Polar Lipids, Alabaster, AL, USA), sphingomyelin (d18:1 with N-acylated 13:0, 17:0, 25:0, semi-synthesized as described in^39^), D6-cholesterol (Cambridge Isotope Laboratory), phosphatidylinositol (16:0/ 16:0 and 17:0/20:4; Avanti Polar Lipids, Alabaster, AL, USA), phosphatidic acid (21:0/22:6, Avanti Polar Lipids, Alabaster AL, USA), phosphatidylethanolamine, phosphatidylserine and phosphatidylglycerol (all 14:1/14:1, 20:1/20:1, 22:1/22:1, semi-synthesized^39^), diacylglycerol (17:0/17:0, Larodan), cholesterol ester (9:0, 19:0, 24:1, Sigma-Aldrich, St. Louis, MO, USA), triacylglycerol (D5- Mix, LM-6000/D5–17:0,17:1,17:1; Avanti Polar Lipids, Alabaster, AL, USA), ceramide and glucosylceramide (both d18:1 with N-acylated 15:0, 17:0, 25:0, semi-synthesized as described in^39^), lactosylceramide (d18:1 with N-acylated C12 fatty acid; Avanti Polar Lipids, Alabaster, AL, USA), (21:0/22:6; Avanti Polar Lipids, Alabaster, AL, USA), and lyso-phosphatidylcholine (17:1; Avanti Polar Lipids, Alabaster, AL, USA). Phosphatidylethanolamine plasmalogen (PE P-)-containing standard mix was supplemented with PE P-Mix 1 (16:0p/15:0, 16:0p/19:0, 16:0p/ 25:0), PE P-Mix 2 (18:0p/15:0, 18:0p/19:0, 18:0p/25:0), and PE P-Mix 3 (18:1p/15:0, 18:1p/19:0, 18:1p/25:0). Semi-synthesis of PE P- was performed as described in^53^. Typically, about 1.5-2.5 nnmol of total lipid was subjected to extraction in the presence of an internal standard mixture containing 50 pmol PC (25 pmol for HIV-1 particles); 50 pmol SM; 25 pmol PE, PS, PI; 20 pmol DAG, TAG, CE; 10 pmol PG, PA, HexCer, Hex2Cer, LPC; 5 pmol Cer, 100 pmol Chol (50 pmol for HIV-1 particles). For quantification of plasmalogens 100 pmol (75 pmol for HIV-1 particles) of a PE P mix was used, containing 16:0p, 18:0p and 18:1p standards in a ratio of 1:1.4:2. Lipid extracts were resuspended in 60 µl methanol and samples were analyzed on an AB SCIEX QTRAP 6500 + mass spectrometer (Sciex, Framingham, MA, USA) with chip-based (HD-D ESI Chip; Advion Biosciences, Ithaca, NY, USA) nano-electrospray infusion and ionization via a Triversa Nanomate (Advion Biosciences, Ithaca, NY, USA) as previously described^39^. Resuspended lipid extracts were diluted 1:10 in 96-well plates (Eppendorf twin tec 96, colorless, Z651400-25A; Sigma-Aldrich, St. Louis, MO, USA) prior to measurement. Lipid classes were analyzed in positive ion mode applying either specific precursor ion (PC, lyso- PC, SM, cholesterol, Cer, HexCer, Hex2Cer, and PE-P) or neutral loss (PE, PS, PI, PG, and PA) scanning as described in^39^.

Data evaluation was performed using LipidView (RRID: SCR_017003; Sciex, Framingham, MA, USA) and an in-house-developed software (ShinyLipids).

### Lipid extraction, derivatization and analysis of phosphoinositides

720 µl of CHCl_3_:MeOH 1:2 were added to all TCA pellets and vortexed for 10 min at RT. Samples were centrifuged at max. speed for 5 min at RT. The supernatants were transferred into fresh tubes on ice, and regarded as neutral extracts, to which the ISD mix was spiked only after they were transferred into fresh tubes. Remaining pellets were stored under argon at – 80 °C for acidic extraction.

For neutral extraction, 10 µl phosphatidylinositol lipid analytical internal standard (ISD) mix (see below for composition) was added to the supernatants, samples were vortexed and spun down briefly. 6 µl of ice cold 12.1 M HCl were added and samples were vortexed immediately within one second of addition for 10 min at 4 °C. 720 µl CHCl_3_ were added to the samples and tubes were vortexed for 5 min at 4 °C, followed by addition of 354 µl 1 M HCl and subsequent vortexing for 2 min at 4 °C. Samples were centrifuged at 1000 g for 5 min. The lower phase was transferred into fresh tubes and dried in a refrigerated Centrivap (Labconco, Kansas City, MO, USA), while preventing air condensation. Dried tubes were flushed with argon and stored at – 80 °C.

For acidic extraction, tubes containing the remaining pellets were placed on ice and 10 µl of the ISD mix (see below for composition) were added. 726 µl of CHCl_3_:MeOH:12.1 M HCl 40:80:1 were added and tubes were vortexed for 15 min at 4 °C. 720 µl CHCl_3_ were added and tubes were vortexed for 5 min at 4 °C, followed by addition of 354 µl 1 M HCl and vortexing for 2 min at 4 °C. Samples were centrifuged at 1000 g for 5 min and the lower phase was transferred into fresh tubes. 702 µl of CHCl_3_:MeOH:1.185 M HCl 3:48:47 (theoretical upper phase) were added to the samples and samples were vortexed for 10 s, followed by centrifugation at 1000 g for 3 min at 4 °C. The lower phase was transferred into fresh tubes and samples were dried in a vacuum concentrator. Samples were flushed with argon and stored at – 80 °C.

The dried lipids were derivatized as described previously^41^ (*please be aware of the danger imposed by trimethylsilyl (TMS-)diazomethane*). The primary reaction with TMS-diazomethane occurs by addition of a methyl group to each hydroxyl on the phosphate groups of phosphoinositides. The reaction takes place in the presence of methanol, which acts as proton donor and removes TMS as a methyl ether, after which a reactive intermediate is formed that methylates one hydroxyl on a phosphate group while releasing N_2(g)_ as by-product^54^. Briefly, the dried lipids were redissolved in 100 uL of methanol:dichloromethane:TMS-diazomethane (2.0 M solution in Et_2_O) (4:5:1) by vortexing. This resulted in a final TMS-diazomethane concentration of 0.2 M. The permethylation reaction started as soon as the solution was added, and the reaction was continued for 40 min at RT. Samples were dried in a CentriVap (Labconco) with a carefully set vacuum control. The dried samples were flushed with argon and stored at − 80 °C prior to mass spectrometric analysis.

The dried, derivatized samples were redissolved in methanol, and quantified by targeted analysis as described previously^41^. Briefly, dried samples were initially re-suspended in 20–100 µl 100% methanol (LC-MS Optima grade, Fisher) prior to chromatographic separation at ambient temperature using a C4 column (Waters Acquity UPLC Protein BEH C4, 1.7 µm 1.1 × 100; 300 A). A Waters Acquity FTN autosampler set at 4 °C injected 1–3 µl of sample via Waters Acquity UPLC. For chromatography of phosphoinositides the mobile phase was delivered over an 18.5 min runtime at a flow rate of 0.1 ml/min by a Waters Acquity UPLC. The gradient was initiated with 10 mM formic acid in water/10 mM formic acid in acetonitrile (37:63 v/v), held for 2 min, then increased to 15:85, v/v in 10 min, then increased to 100% B and held at 100% for 2.8 min prior to 3 min re-equilibration to starting conditions. The gradient was initiated with 10 mM formic acid in water/10 mM formic acid in acetonitrile (45:55 v/v), held for 2 min, then increased to 15:85, v/v in 10 min, then increased to 100% B and held at 100% for 1.8 min and then re-equilibrated to starting conditions for 3 min.

The lipid analytical internal standards were ammonium salts of 1-heptadecanoyl-2-(5Z,8Z, 11Z,14Z-eicosatetraenoyl)-sn-glycero-3-phospho-(1′-myo-inositol 3′,4′,5′-trisphosphate) [17:0, 20:4 PI(3,4,5)P_3_]; 1-heptadecanoyl-2-(5Z,8Z,11Z,14Z-eicosatetraenoyl)-sn-glycero-3-phospho-(1′-myo-inositol 4′,5′-bisphosphate) [17:0, 20:4 PI(4,5)P_2_]; 1-heptadecanoyl-2-(5Z,8Z,11Z,14Z-eicosatetraenoyl)-sn-glycero-3-phospho-(1′-myo-inositol 4′-phosphate) [17:0, 20:4 PI(4)P]; and 1-heptadecanoyl-2-(5Z,8Z,11Z,14Z-eicosatetraenoyl)-sn-glycero-3-phospho-(1′-myo-inositol) [17:0, 20:4 PI] from Avanti Polar Lipids (LIPID MAPS MS Standards; Avanti Polar Lipids, Alabaster, AL, USA). PIP, PIP_2_ and PIP_3_ standards were dissolved at 100 ng/µl in CHCl_3_:CH_3_OH:H_2_O 20:9:1. The standards were combined, dried and re-dissolved in 1 ml of CHCl_3_:CH_3_OH:H_2_O 20:9:1 to give the following final concentrations: 4 ng/µl PI, 2 ng/µl PIP, 2 ng/µl PIP_2_, 0.2 ng/µl PIP_3_.

Peak areas for lipid species and standards were quantified by integrating curves using Waters’ MassLynx MS-Software V4.1 (RRID:SCR_014271; Waters Corporation, Milford, MA, USA). For absolute calibrations and comparisons of different samples, peak areas of endogenous species were normalized to those of the corresponding internal standards described above.

### Thin-layer chromatography analysis of phosphoinositides

Dried lipid extracts of phosphoinositides were resuspended in chloroform:methanol (2:1) and applied onto a 10 × 10 cm silica HPTLC F254 plate. Plates were pre-coated by soaking them into a mixture of methanol/water (2/3) containing 1% potassium oxalate (w/v) for 30 min, air dried in a well-ventilated hood and activated at 100 °C for 1 hour. Plates were developed in CHCl_3_/C_3_H_6_O/MeOH/AcOH/H_2_O (80/30/26/24/14) for 9 cm and let dry. For detection, plates were exposed to iodine vapors, and imaged under UV light using a BioRad Chemidoc Touch System (BioRad, Herkules, CA, USA).

### Statistical analysis

Statistical analysis was performed with supervision of the Heidelberg Hospital Medical Biometry and Informatics (IMBI) Department. Statistics are given as mean ± SD. Student’s t-test was used to test for statistical significance. P-values are given in figure legends.

## Supplementary material

Supplementary information
