# Supplementary material for: Quantification of phosphoinositides reveals strong enrichment of PIP2 in HIV-1 compared to producer cell membranes
Source: Sci Rep. 2019 Nov 27;9:17661. doi: 10.1038/s41598-019-53939-z (PMC6881329; doi:10.1038/s41598-019-53939-z)
Supplement: Supplementary file 1 — Supplementary information [file 41598_2019_53939_MOESM1_ESM.pdf]

Supplementary Information

Quantification of phosphoinositides reveals strong enrichment of PIP_2 in HIV-1 compared to producer cell membranes

Frauke Mücksch[1*], Mevlut Citir[2*], Christian Lüchtenborg[3], Bärbel Glass[1], Alexis Traynor-Kaplan[4,5], Carsten Schultz[2,6], Britta Brügger[3], and Hans-Georg Kräusslich[1,7]

Supplementary Table 1 – Lipid composition and lipid species distribution in HIV-1 membranes, PM and cells

1) Lipid composition (in mol% of total lipids measured) of HIV-1 particles (OP), PM preparations from uninfected (PM) or infected (PM inf) cells and whole uninfected (cells) and infected (cells inf) cells. Shown are values from n = 4 independent virus productions and n = 3 independent PM purifications / cell isolations.

2)-25) Molecular species distribution of the respective lipid given as mol% of total lipids measured. Shown are values from n = 4 independent virus productions and n = 3 independent PM purifications / cell isolations.

Supplementary Table 2 – Lipid molecule numbers per average HIV-1 particle

Molecule numbers per average HIV-1 particle for individual lipid classes for n=4 independent virus preparations (OP 1 to OP 4).

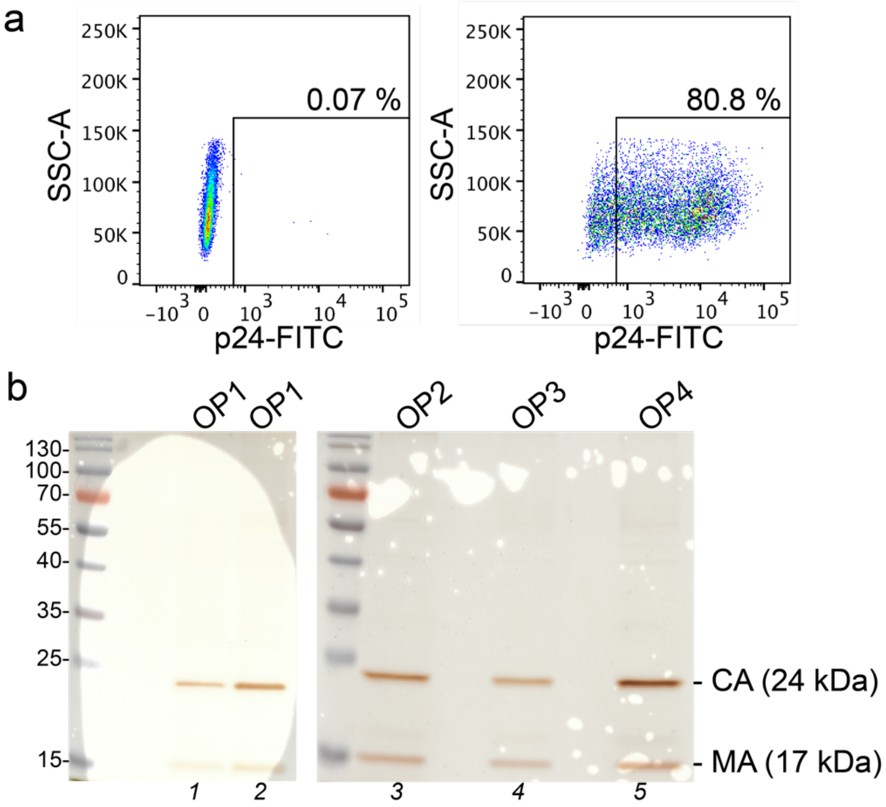

c

	ng CA/µl (ELISA)	genomes/µl
OP 1	411	$2.44*10^9$
OP 2	105	$1.88*10^9$
OP 3	76	$9.9*10^8$
OP 4	101	$1.6*10^9$

Supplementary Figure S1 – Virus characterization

(a) Representative FACS-plot of HIV-1 infected MT-4 cell cultures analyzed for CA expression by immunostaining. Gates were set according to uninfected, stained sample (left panel). Infected sample (right panel) shows 80.8% HIV-1 infected cells in this representative experiment. **(b)** Four independent Optiprep gradient purified HIV-1 particle preparations from infected MT-4 cells (OP1-OP4) were separated on 12.5 % SDS gels. Virion-associated proteins were visualized by silver staining according to standard procedures, showing MA and CA containing Gag derivatives (as indicated on the right). Positions of molecular mass standards (in kilodaltons) are shown on the left. Gels were loaded with 0.5 µl (lane 1), 1 µl (lane 2) or 2 µl (lanes 3-5) of the indicated particle preparations. **(c)** CA content by ELISA (column 1) and vRNA genome copy numbers (column 2) in HIV-1 purifications OP 1 to OP 4.

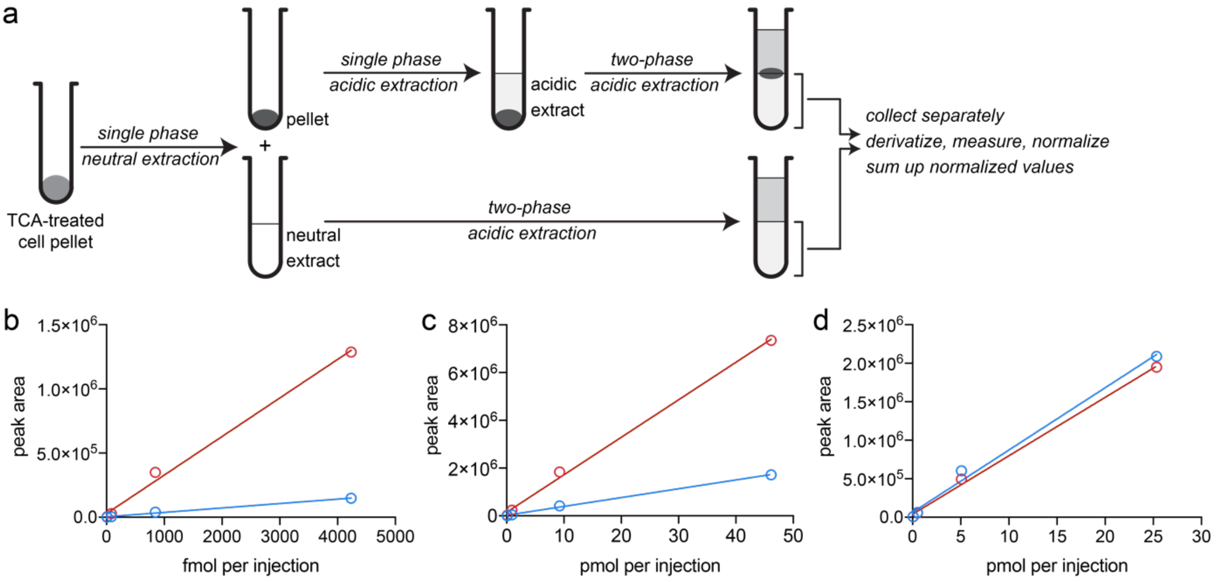

e

	acidic	neutral
PC	0.25 ± 0.04	99.75 ± 0.04
PI	30.62 ± 1.88	69.38 ± 1.88
PIP	88.01 ± 0.71	11.99 ± 0.71
PIP2	94.57 ± 0.17	5.43 ± 0.17
PIP3	100	0

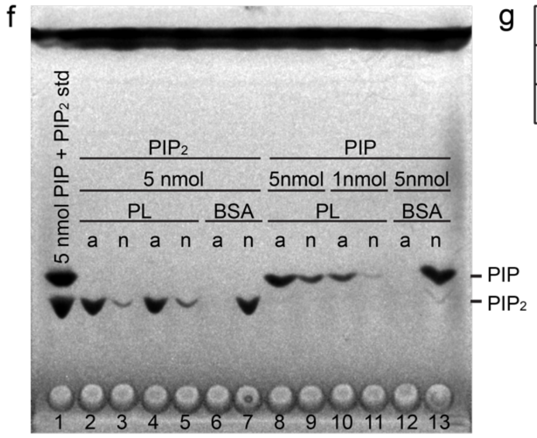

g

	acidic	neutral
PIP2	92.64	7.36
PIP	75.51	24.49

Supplementary Figure S2 – Lipid extraction for phosphoinositide analysis

(a) Schematic of sequential two-phase extraction; for details see Materials & Methods. Titration curves for neutral (blue) and acidic (red) extracts of PIP_3 37:4 ($R^2_{neutral}$=0.9957; R^2_{acidic}=0.9944) **(b)**, PIP_2 37:4 ($R^2_{neutral}$=0.9981; R^2_{acidic}=0.9976) **(c)** and PIP 37:4 ($R^2_{neutral}$=0.9919; R^2_{acidic}=0.9971) **(d)** standards used in all further experiments. **(e)** Percent recovery of the respective lipid in the acidic and neutral extract; data are from triplicate experiments ± standard deviation. **(f)** The indicated amounts of PIP_2 or PIP were TCA precipitated in the presence of PL or BSA and precipitates were subjected to two-step neutral (n) and acidic (a) lipid. Extracts were subjected to TLC separation and stained with iodine vapors. **(g)** Percent recovery of PIP_2 and PIP after TCA precipitation in the presence of PL were calculated from integrated intensities of the respective bands in panel (e) after background subtraction in ImageJ.

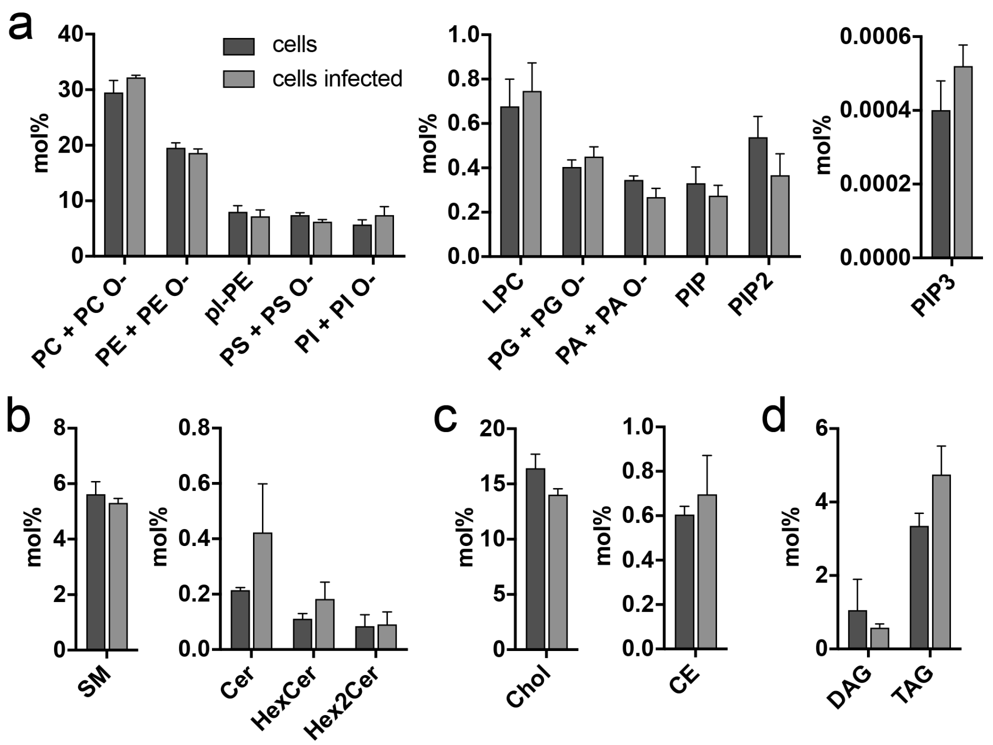

Supplementary Figure S3 – Lipid composition of whole cell membranes from uninfected and HIV–1 infected MT-4 cells.

Lipid composition (in mol% of total lipids) of uninfected (dark grey) or HIV-1 infected (medium grey) MT-4 cells. Shown are phospholipids **(a)** with an expanded scale for minor lipid species in middle and right panels, sphingolipids **(b)** and sterols **(c)** with an expanded scale for minor lipids in right panels, and glycerolipids **(d)**. Data represent mean values and standard deviation of n = 3 independent experiments. PE O-, PS O-, PI O-, PG O-, and PA O- species contain either an ether linked alcohol or an odd-chain fatty acyl moiety.

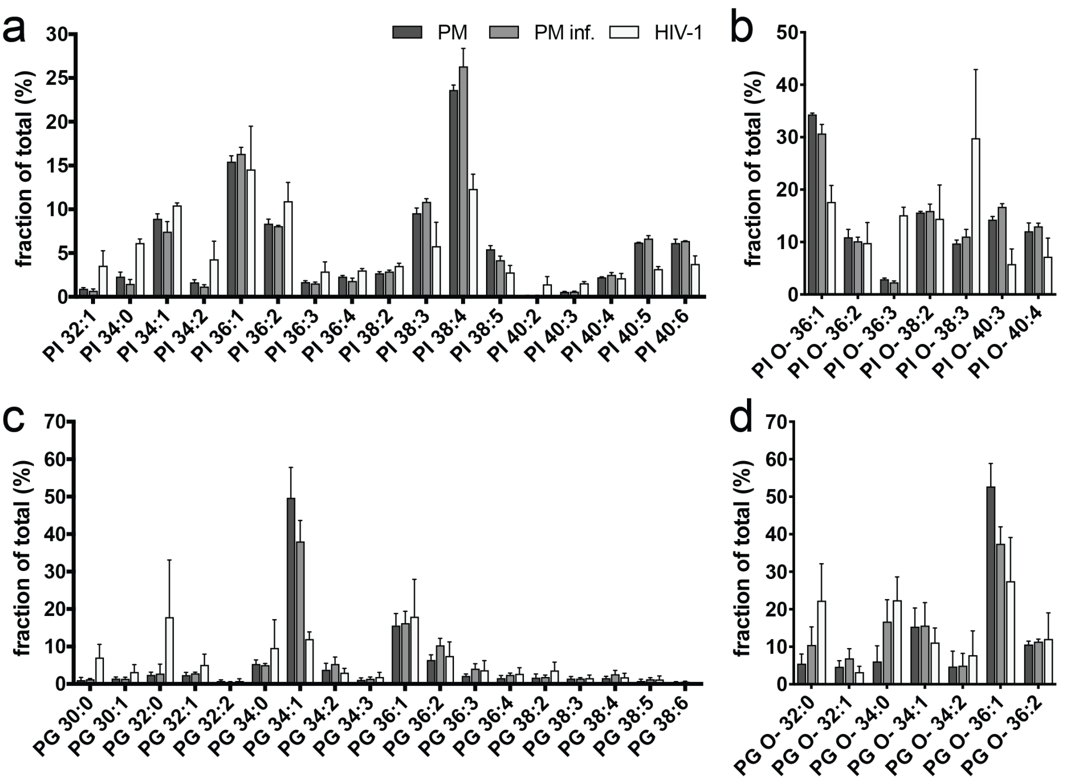

Supplementary Figure S4 – Molecular species distribution of phosphatidylinositol (PI) and phosphatidylglycerol (PG).

Quantitative lipid analysis of PM isolations from uninfected (PM) and infected (PM inf.) MT-4 cells and viral membranes (HIV) was performed as described in materials and methods. Molecular species distribution of PI **(a)**, ether-PI (PI O^-) **(b),** PG **(c)** and ether-PG (PG O^-) **(D)** is given as fraction of total. Data represent mean values and standard deviation of n=3 (PM isolations) or n=4 (virus purifications) independent experiments.

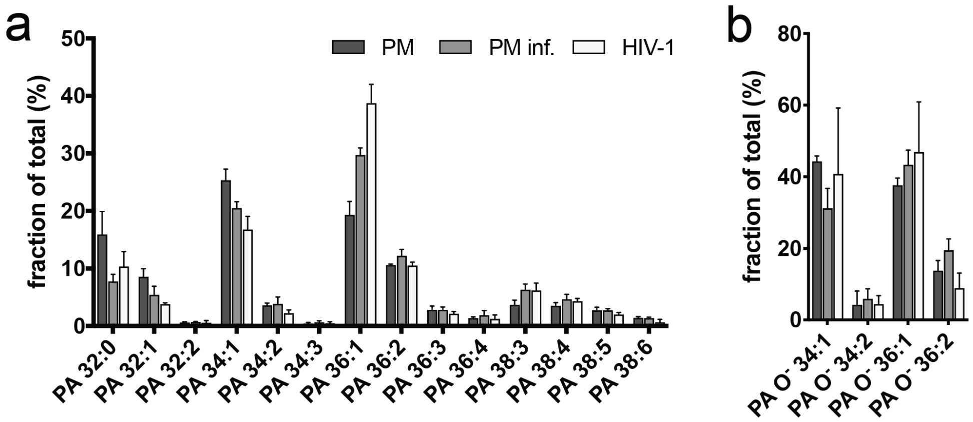

Supplementary Figure S5 – Molecular species distribution of phosphatidic acid (PA).

Quantitative lipid analysis of PM isolations from uninfected (PM) and infected (PM inf.) MT-4 cells and viral membranes (HIV) was performed as described in materials and methods. Molecular species distribution of PA **(a)** and ether-PA (PA O^{-}) **(b)** is given as fraction of total. Data represent mean values and standard deviation of n=3 (PM isolations) or n=4 (virus purifications) independent experiments.

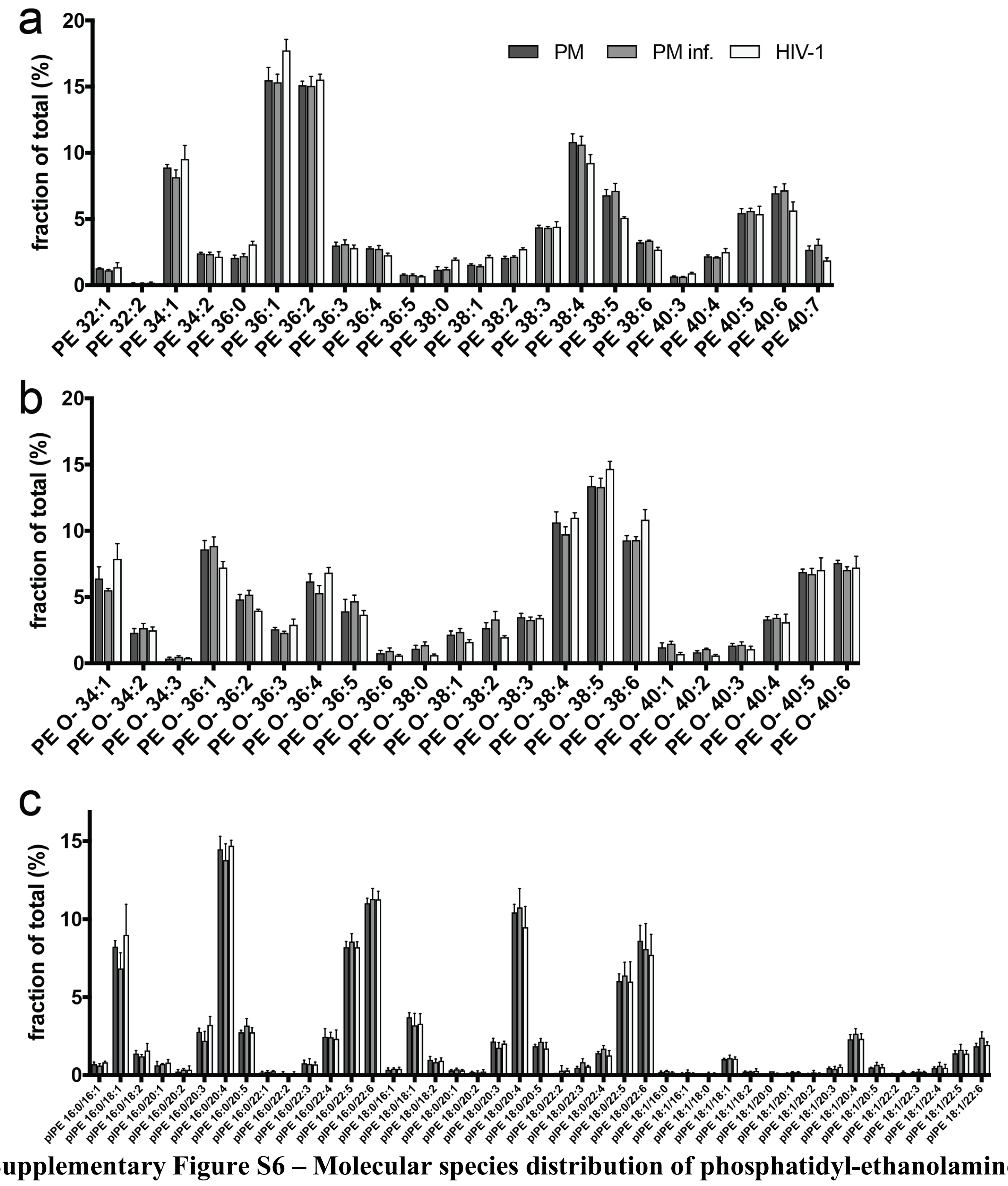

Supplementary Figure S6 – Molecular species distribution of phosphatidyl-ethanolamine (PE).

Quantitative lipid analysis of PM isolations from uninfected (PM) and infected (PM inf.) MT-4 cells and viral membranes (HIV) was performed as described in materials and methods. Molecular species distribution of PE **(a)**, ether-PE (PE O^-) **(b)** and plasmalogen-PE (plPE) **(c)** is given as fraction of total. Data represent mean values and standard deviation of n=3 (PM isolations) or n=4 (virus purifications) independent experiments.

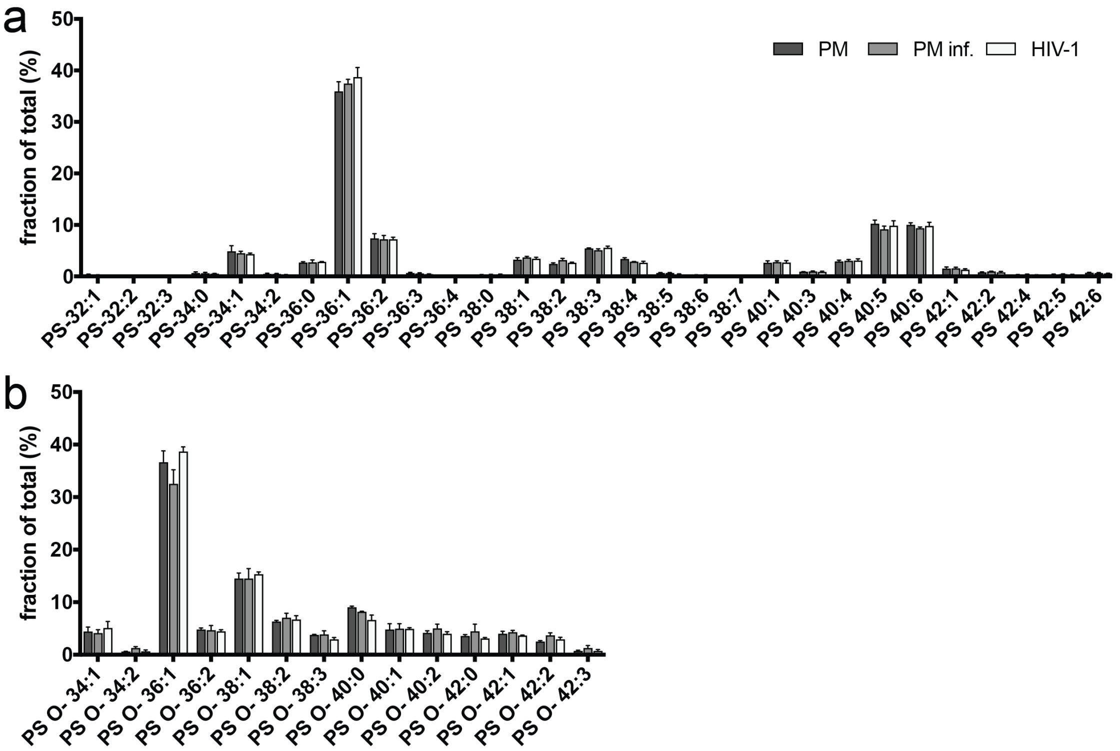

Supplementary Figure S7 – Molecular species distribution of phosphatidylserine (PS).

Quantitative lipid analysis of PM isolations from uninfected (PM) and infected (PM inf.) MT-4 cells and viral membranes (HIV) was performed as described in materials and methods. Molecular species distribution of PS **(a)** and ether-PS (PS O^-) **(b)** is given as fraction of total. Data represent mean values and standard deviation of n=3 (PM isolations) or n=4 (virus purifications) independent experiments.

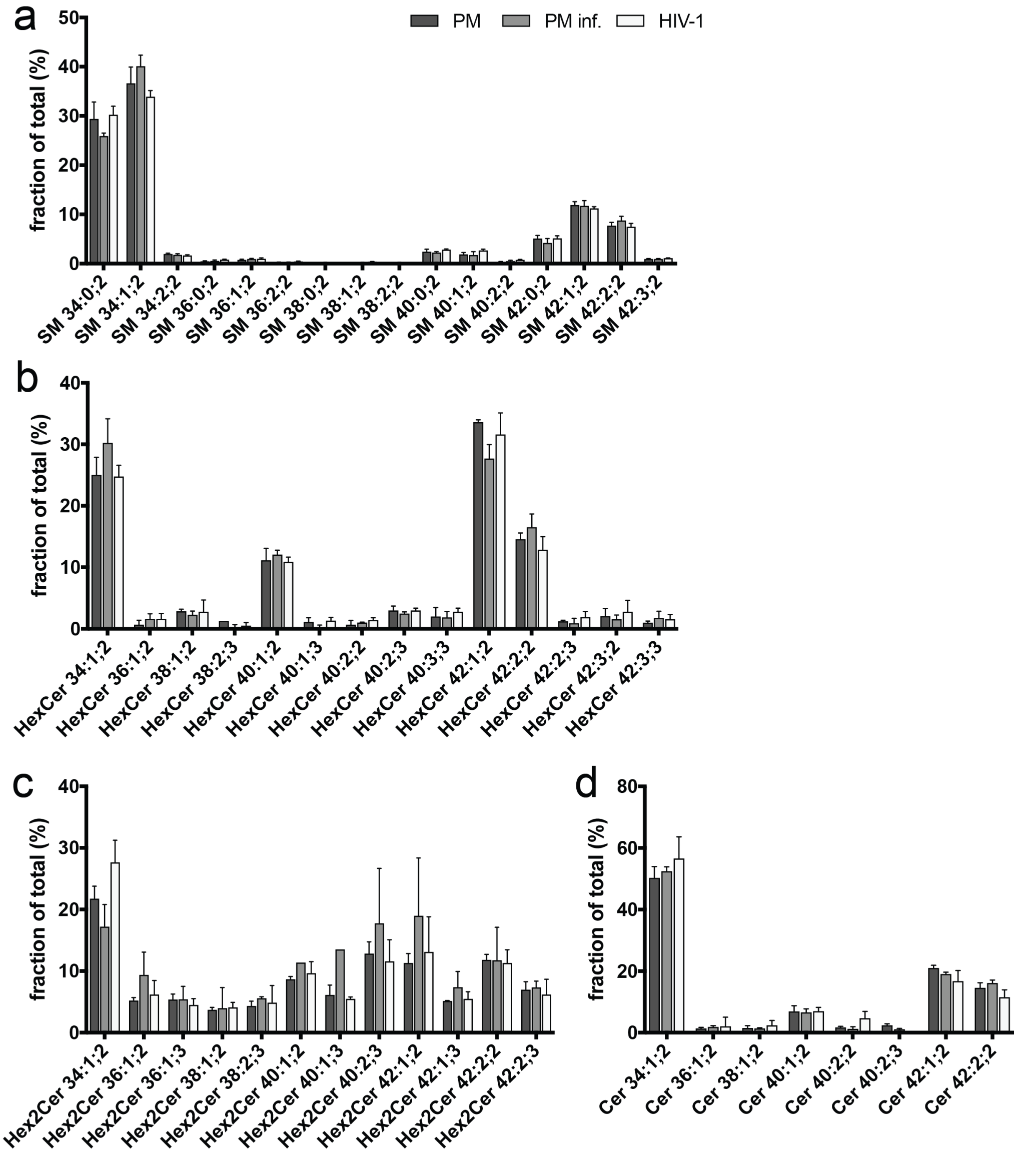

Supplementary Figure S8 – Molecular species distribution of sphingolipids.

Quantitative lipid analysis of PM isolations from uninfected (PM) and infected (PM inf.) MT-4 cells and viral membranes (HIV) was performed as described in materials and methods. Molecular species distribution of SM **(a)**, HexCer **(b),** Hex2Cer **(c)** and Cer **(d)** is given as fraction of total. Data represent mean values and standard deviation of n=3 (PM isolations) or n=4 (virus purifications) independent experiments.

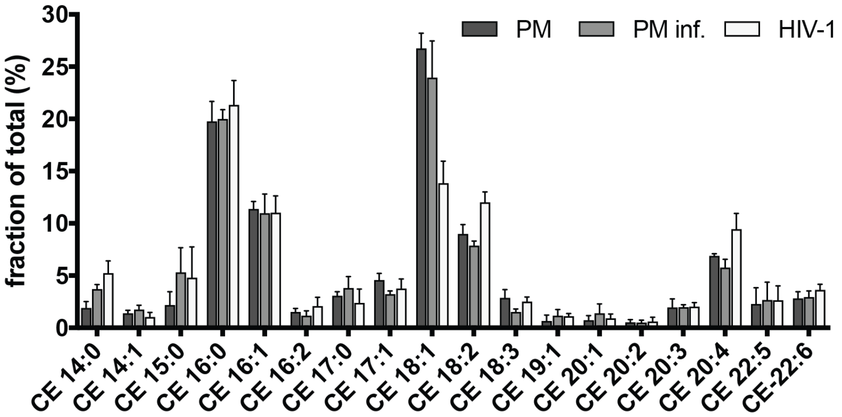

Supplementary Figure S9 – Molecular species distribution of cholesterol esters.

Quantitative lipid analysis of PM isolations from uninfected (PM) and infected (PM inf.) MT-4 cells and viral membranes (HIV) was performed as described in materials and methods. Molecular species distribution is given as fraction of total. Data represent mean values and standard deviation of n=3 (PM isolations) or n=4 (virus purifications) independent experiments.
